# Supplementary material for: Hide and seek shark teeth in Random Forests: machine learning applied to Scyliorhinus canicula populations
Source: PeerJ. 2022 Jul 4;10:e13575. doi: 10.7717/peerj.13575 (PMC9261926; doi:10.7717/peerj.13575)
Supplement: Supplemental Information 1 — x, y, and z are the 3D dimensions. L, landmark; SL, semilandmark. [file peerj-10-13575-s001.pdf]

ADDITIONAL FILE 1

Point	x	y	z
L-1	0.5	1.5	0.2
SL-2	0.3	0.2	0.1
SL-3	0.4	0.1	0.3
SL-4	0.3	0.1	0.6
SL-5	0.3	0.1	0.2
SL-6	0.2	0.2	0.1
SL-7	0.1	0.2	0.1
SL-8	0.1	0.1	0.1
SL-9	0.1	0.2	0.1
SL-10	0.5	0.2	0.1
SL-11	4.5	0.3	0.1
SL-12	1.0	1.3	0.1
L-13	0.4	1.5	0.2
SL-14	0.2	0.2	0.2
L-15	0.4	0.6	0.8
SL-16	0.5	0.3	0.4
L-17	0.2	0.4	0.4
SL-18	0.2	0.2	0.4
L-19	1.5	0.6	0.1
SL-20	0.3	0.7	0.2
L-21	0.4	0.8	0.2
SL-22	0.1	1.0	0.2
SL-23	0.7	1.1	0.1
SL-24	1.5	1.4	0.1
SL-25	0.9	0.9	0.1
SL-26	0.3	0.1	0.1
SL-27	0.2	0.2	0.1
SL-28	0.5	0.5	0.1
SL-29	0.2	0.2	0.2
SL-30	0.1	0.2	0.3
SL-31	0.1	0.1	0.2
SL-32	0.1	0.2	0.2
L-33	0.3	0.3	0.2
SL-34	0.4	0.4	0.1
SL-35	0.4	0.6	0.2
SL-36	0.2	0.2	0.7
SL-37	0.2	0.2	0.3
SL-38	0.3	0.5	0.2

Mean Decrease Accuracy values in % with tooth shape data. x, y, and z are the 3D dimensions. L, landmark; SL, semilandmark.
