# Supplementary material for: Hide and seek shark teeth in Random Forests: machine learning applied to Scyliorhinus canicula populations
Source: PeerJ. 2022 Jul 4;10:e13575. doi: 10.7717/peerj.13575 (PMC9261926; doi:10.7717/peerj.13575)
Supplement: Supplemental Information 2 — x, y, and z are the 3D dimensions. L, landmark; SL, semilandmark. [file peerj-10-13575-s002.pdf]

ADDITIONAL FILE 2

Point	x	y	z
L-1	0.1	1.0	0.2
S-L2	0.1	0.2	0.1
SL-3	0.3	0.1	0.2
SL-4	0.2	0.1	0.2
SL-5	0.1	0.2	0.2
SL-6	0.1	0.3	0.2
SL-7	0.1	0.2	0.2
SL-8	0.1	0.1	0.2
SL-9	0.1	0.1	0.2
SL-10	0.3	0.3	0.2
SL-11	5.7	1.6	0.3
SL-12	0.6	1.2	0.2
L-13	0.2	1.0	0.6
SL-14	0.2	0.2	0.3
L-15	0.9	0.7	0.8
SL-16	0.5	0.3	0.4
L-17	0.3	0.5	0.6
SL-18	0.2	0.1	0.2
L-19	1.1	1.8	0.1
SL-20	0.2	0.3	0.2
L-21	0.3	0.6	0.1
SL-22	0.2	0.8	0.2
SL-23	1.7	0.8	0.2
SL-24	2.1	0.7	0.1
SL-25	0.4	0.2	0.1
SL-26	0.2	0.1	0.1
SL-27	0.3	0.3	0.1
SL-28	0.5	0.7	0.1
SL-29	0.2	0.2	0.1
SL-30	0.1	0.1	0.1
SL-31	0.2	0.1	0.1
SL-32	0.6	0.1	0.1
L-33	0.7	0.4	0.2
SL-34	0.2	0.3	0.2
SL-35	0.2	0.6	0.2
SL-36	0.1	0.2	0.6
SL-37	0.4	0.2	0.6
SL-38	0.2	0.4	1.0

Mean Decrease Accuracy values in % with tooth form data. x, y, and z are the 3D dimensions. L, landmark; SL, semilandmark.
